# Lipid profile in girls with precocious puberty: a systematic review and meta-analysis

**DOI:** 10.1186/s12902-023-01470-8

**Published:** 2023-10-18

**Authors:** Mei Jiang, Ying Gao, Kai Wang, Ling Huang

**Affiliations:** 1 Beijing Research Institute of Chinese Medicine, Beijing University of Chinese Medicine, Beijing, China; 2 Department of Acupuncture, Wangjing Hospital of China Academy of Chinese Medical Sciences, Beijing, China; 3 School of Chinese Materia Medica, Beijing University of Chinese Medicine, Beijing, China; 4 School of Traditional Chinese Medicine, Beijing University of Chinese Medicine, No.11 Beisanhuandong Road, Chaoyang District, Beijing, 100029 P. R. China

**Keywords:** Precocious puberty, Lipids, Cholesterol, LDL cholesterol, HDL cholesterol, Dyslipidemias, Cardio vascular disease

## Abstract

**Objective:**

Many studies have investigated the impact of precocious puberty on cardiovascular disease (CVD) outcomes and the association between lipid profile levels and precocious puberty. However, the results have been inconsistent. The aim of this meta-analysis was to evaluate whether triglyceride (TG), total cholesterol (TC), high density lipoprotein (HDL)and low density lipoprotein (LDL) levels were altered in girls with precocious puberty compared with healthy controls.

**Methods:**

References published before June 2022 in the EMBASE, Cochrane Library, PubMed and Web of Science databases were searched to identify eligible studies. A DerSimonian-Laird random-effects model was used to evaluate the overall standard mean difference (SMD) between precocious puberty and healthy controls. Subgroup analyses and sensitivity analyses were preformed, and publication bias was assessed.

**Results:**

A total of 14 studies featuring 1023 girls with precocious puberty and 806 healthy girls were selected for analysis. The meta-analysis showed that TG (SMD: 0.28; 95% CI: 0.01 to 0.55; *P* = 0.04), TC (SMD: 0.30; 95% CI: 0.01 to 0.59; *P =* 0.04), LDL (SMD: 0.45; 95% CI: 0.07 to 0.84; *P =* 0.02)levels were significantly elevated in girls with precocious puberty. HDL levels did not change significantly (SMD: -0.06; 95% CI: -0.12 to 0.61; *P =* 0.62). Subgroup analyses revealed that the heterogeneity in the association between lipid profile and precocious puberty in this meta-analysis may arise from disease type, region, sample size, chronological age, body mass index difference and drug usage.

**Conclusion:**

Lipid profile levels altered in girls with precocious puberty compared with healthy controls. In order to minimize the risk of CVD morbidity and mortality, early interventions were needed to prevent obesity in children and adolescents, especially those with precocious puberty.

**Supplementary Information:**

The online version contains supplementary material available at 10.1186/s12902-023-01470-8.

## Introduction

Precocious puberty is defined as the development of secondary sexual characteristics in girls by the age of 8 years and in boys by the age of 9 years [1]. A growing percentage of girls are going through precocious puberty [2, 3]. According to whether the hypothalamic-pituitary-gonadal axis (HPGA) occurs or not, precocious puberty is classified as central precocious puberty (CPP) with the HPGA occurring driven by early increased gonadotropin-releasing hormone (GnRH) secretion, which accounts for roughly 80% [4, 5], and as peripheral precocious puberty (PPP) which is independent of GnRH secretion, as well as incomplete precocity, which is the variation of CPP, including premature pubarche (PP), premature adrenarche (PA), premature thelarche (PT) and premature menarche [6–9]. PT is caused by transient partial activation of HPGA and overproduction of follicle stimulating hormone (FSH) and is characterized by isolated breast development without any other signs of sexual maturation [10]. However, 13% of PT cases may develop into CPP [11]. PA is referred to as an increase of adrenal androgen level independent of the HPGA, it is identified as pubarche including the presence of pubic and axillary hair, apocrine body odor and acne by age 8 for girls and age 9 for boys [12].

In girls, early maturational timing is linked to increased all-cause, cancer, and cardiovascular disease (CVD) [13, 14]. Accordingly, an earlier age of menarche is linked to a higher risk of obesity [15–18], hypertension [13], type 2 diabetes [18], ischemic heart disease, and stroke occurrences [13], as well as other conditions in later life. According to a recent study, high childhood obesity may be to blame for the effects of premature menarche on cardiovascular risk in adults [19]. In accordance, high adiposity during adolescence predicts premature menarche [20, 21]. On the other hand, women who have a history of early menarche have greater increases in adiposity during adolescence and in the early years of adulthood [15, 20]. As a result, it does appear that early maturation plays a role in unfavorable metabolic programming.

There are conflicting reports about the effects of precocious puberty on lipid metabolism so far. Ibáñez et al. [22]. found the serum levels of triglyceride (TG), total cholesterol (TC) and low density lipoprotein (LDL) increased, and the level of high density lipoprotein (HDL) decreased in girls with premature pubarche.And similar findings were found in several cohort studies [23, 24]. In contrast, no relationship between early maturational timing and lipid profile has been found in other studies [25–32].

Hence, understanding the potential link between lipid profile and precocious puberty has public health implications for reducing the risk of cardiovascular morbidity and mortality in women. Precocious puberty may provide important information to unravel the effects of pubertal onset and age on lipid profile levels. Therefore, the aim of this systematic review and meta-analysis was to assess lipid profile levels in girls with precocious puberty and healthy controls.

## Materials and methods

### Reporting guidelines

This systematic review and meta-analysis was designed according to the Preferred Reporting Items for Systematic Reviews and Meta-Analyses (PRISMA) statement [33], and was prospectively registered on PROSPERO (registration number: CRD42022357819). This study was conducted according to the guidelines for Meta-analysis of Observational Studies in Epidemiology (MOOSE) [34].

### Search strategy

The population/intervention/comparison/outcome (PICO) components were as follows: P (girls before the age of 10), I (girls with precocious puberty), C (healthy girls), O (serum levels of TG, TC, HDL and LDL).To identify eligible studies, an exhaustive literature search was conducted in the Cochrane Library, PubMed, EMBASE and Web of Science databases (without restricting by language, location and journal) through June 2022 to identify published research using the following keywords: “precocious puberty” OR “sexual precocity” OR “premature puberty” OR “precocious sexual maturation” OR “early puberty” OR “earlier puberty” OR “early pubertal timing” OR “early maturation” OR “isolated premature thelarche” OR “premature thelarche” OR “premature pubarche” OR “premature adrenarche” OR “premature menarche” OR “early age of menarche” OR “PP” OR “CPP” OR “PPP” OR “IPT” OR “PT”AND “lipid profile” OR “total cholesterol” OR “triacylglycerol” OR “triglyceride” OR “triglycerides” OR “high-density lipoprotein” OR “low-density lipoprotein” OR “TC” OR “TG” OR “HDL” OR “LDL” OR “HDL-C” OR “LDL-C”.

### Inclusion and exclusion criteria

The inclusion criteria for eligible studies were as follows:(1) original observational study of humans; (2) all patients involved in studies were diagnosed with precocious puberty, including CPP, PP, PA and PT before 8 years old; (3) all subjects involved in studies were younger than 10 years old; (4) studies focusing on the association between the serum TG, TC, HDL, LDL levels and precocious puberty; and (5) studies included data on the lipid profile levels for patients with precocious puberty and healthy age-matched prepubertal girls.

The exclusion criteria were as follows: (1) laboratory or animals studies; (2) reviews or case reports; (3) duplicate publications; (4) subjects involved in studies were diagnosed with sex hormone releasing tumors and the Cushing’s Syndrome; (5) subjects participating in studies were diagnosed with diabetes, thyroid dysfunction or hyperprolactinemia; (6) studies without healthy control groups; and (7) studies that did not provide definitive data on serum TG/TC/HDL/LDL levels.

### Study selection and data extraction

**Fig. 1:**
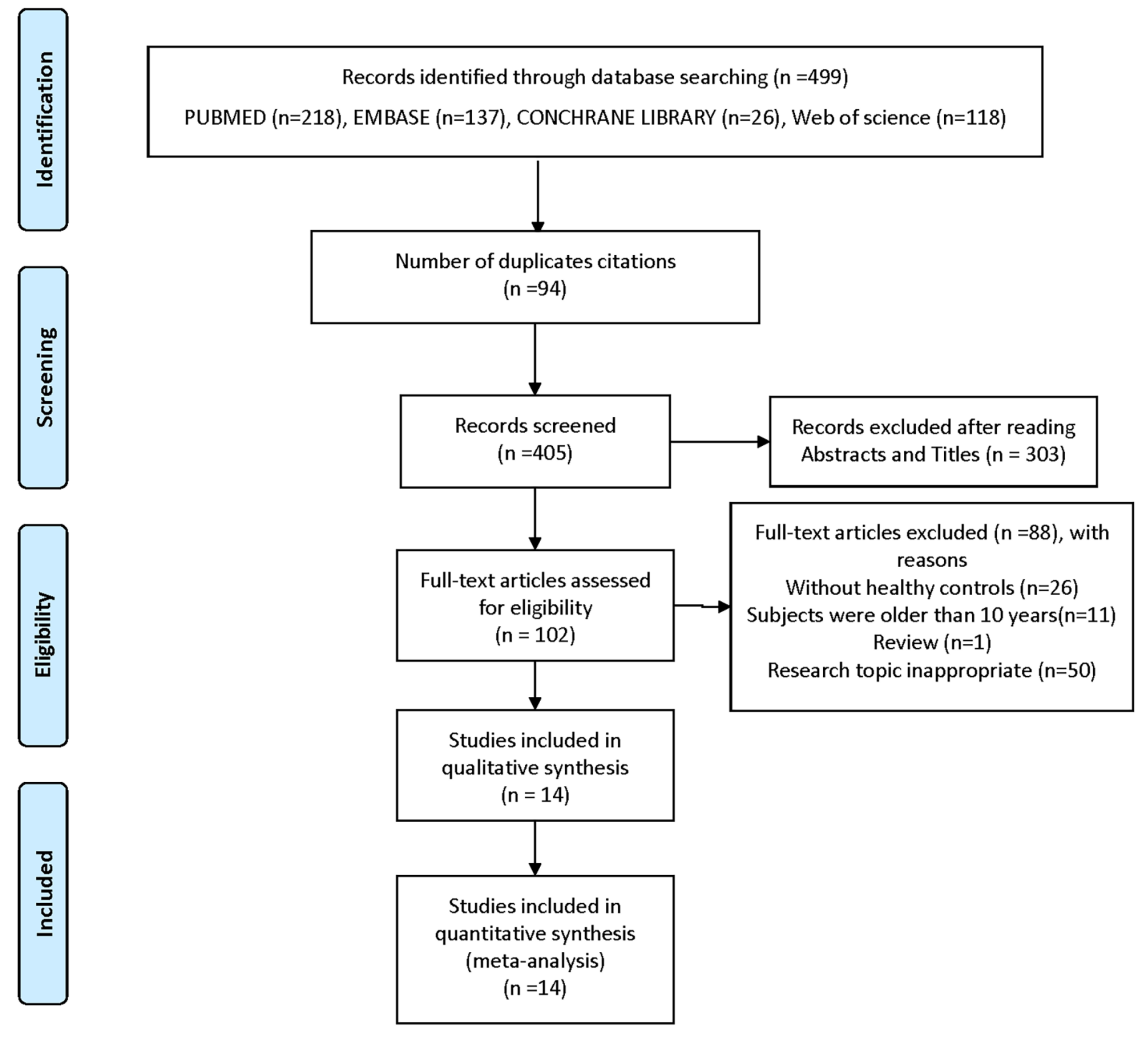
Flow chart of the selection process. *From: Moher D, Liberati A, Tetzlaff J, Altman DG, The PRISMA Group (2009). Preferred Reporting Items for Systematic Reviews and Meta-Analyses: The PRISMA Statement. PLoS Med 6(7): e1000097. doi*: 10.1371/journal.pmed1000097

Literature screening and data extraction were done independently by two researchers (MJ and YG). Discrepancies between two reviewers were resolved in consultation with a third reviewer (LH). Firstly, citations were imported into EndNote to identify duplicate citations. Secondly, research titles and abstracts were screened, and those did not meet the inclusion criteria were excluded. Finally, the full literature was read, and the studies were further excluded based on inclusion and exclusion criteria. A flow chart of the PRISMA interpretation the selection process was shown in Fig. 1. TG, TC, HDL and LDL [mean ± standard deviation (SD)] levels were extracted from the literature, and all data were reviewed by LH.

When no continuous variables were reported, each author was contacted and asked to provide the raw data. In cases where contact failed, standard deviation values were calculated from the median, quartiles or ranges or [35, 36], if the data were displayed graphically only, values were estimated by digital ruler software (Getdata Graph Digitizer, version 2.25) [37, 38].

### Quality assessment

The quality of the selected studies was evaluated according to the Newcastle-Ottawa Scale (NOS) star system [39].

### Statistical analysis

A meta-analysis was performed on the data extracted from the included studies. As the available literature was inconsistent in terms of units, the combined standardized mean difference (SMD) and 95% confidence intervals (CI) were used to determine the associations between serum TG, TC, HDL, LDL levels and precocious puberty. The Cochran’s Q two-sided homogeneitytest [40] was used to test for heterogeneity of the studies. A Mantel-Haenszel fixed-effect model was used if I-square (*I*^*2*^) < 50% and conversely, a DerSimonian-Laird random-effects model was used [41, 42]. Subgroup analyses were performed to determine the association between serum TG, TC, HDL, LDL levels and study characteristics to test whether this could explain heterogeneity.Sensitivity analyses were performed to test the robustness of the combined SMD by excluding each study. The funnel plot method (in the case of the number of inclusions was ≥ 10) was used to test for publication bias. All analyses were performed using RevMan software, and differences were considered statistically significant at *P* < 0.05.

## Results

### Literature search

After reading the titles and abstracts of the initial 405 articles, 303 were excluded because they did not meet the inclusion criteria. 102 articles were included for full-text assessment, of which 88 were excluded: 26 without healthy controls; 11 subjects aged 10 years or older; one review; and fifty research topics were inappropriate. A total of 14 eligible papers were included (Fig. 1), all studies were observational in design and included individual data from 1023 cases and 806 healthy controls [22–32, 43–45]. The baseline characteristics, such as author, year, geographical location, study design, sample size, chronological age (CA), bone age (BA), tanner stage, body mass index (BMI), medication usage, measurement method, lipid profile and key findings included in the studies were shown in Table 1.

**Table 1:** Characteristics of studies and participants included in the meta-analysis

No.	Study	Region	Study design	Sample size (n)		Chronological Age(y)		Bone Age		Tanner stage		BMI(kg/m^2^)		Drug used	Measuring method	Primary conclusion
				Cases	Control	Cases	Control	Cases	Control	Cases	Control	Cases	Control			
1.	Ibáñez (1998) [43]	Spain (Barcelona)	cross-sectional study	PP 21	14	7.5 ± 0.2	8.5 ± 0.3	8.6 ± 0.2	7.8 ± 0.3	I	I	18.8 ± 0.6	17.0 ± 0.4	No	CHOD-PAP and GPO-PAP based methods in a Hitachi 917 autoanalyser (Boehringer Mannheim, Mannheim, Germany)	TG increased in PP; TC, HDL, LDL remained unchanged in PP
2.	Ibáñez (2004) [22]	Spain (Barcelona)	cross-sectional study	PP 33	37	8.0 ± 0.57	8.3 ± 1.83	9.1 ± 0.57	NA	I	I	18.5 ± 0.3	17.7 ± 0.4	No	NA	TG and LDL increased in PP; HDL decreased in PP
3.	Teixeira (2004) [25]	Brazil (Rio de Janeiro)	cross-sectional study	PP 25	14	7.3 ± 1.5	6.4 ± 1.3	NA	NA	NA	NA	19.7 ± 4.4	19.3 ± 4.6	NA	TC, HDL and TG were measured by an automated enzymatic colorimetric method; LDL was calculated using the Friedewald formula	TG, TC, HDL and LDL remained unchanged in PP
4.	Guven (2005) [44]	Turkey (Ankara)	cross-sectional study	PA 24	13	7.6 ± 0.1	6.9 ± 0.3	7.6 ± 0.2	6.4 ± 0.4	II	II	17.1 ± 0.33	15.9 ± 0.4	No	TC, TG and HDL were measured by the Abbott-Aeroset system through enzyme assay; LDL was estimated by indirect calculation	TC and LDL increased in PA; TG and HDL remained unchanged in PA
5.	Larqué (2010) [26]	Spain (Granada)	cross-sectional study	PP 22	20	8.34 ± 0.29	9.12 ± 0.37	NA	NA	I	I	19.33 ± 0.71	17.30 ± 0.60	No	Automatic analyzer (Roche-Hitachi Modular PyD Autoanalyzer, Roche Laboratory Systems)	TG, TC, HDL and LDL remained unchanged in PP
6.	Sorensen (2010) [45]	Denmark (Copenhagen)	cross-sectional study	CPP 15	24	8.67 ± 1.96	9.26 ± 3.15	NA	NA	II, III	I	18.9 ± 3.4	16.21 ± 4.18	13 girls with CPP were initiated on long-acting GnRHa treatment with sc injections every 28th day	Enzymatic colorimetric analyses (CFAS TC/LDL/HDL plus, TG GPO-PAP; Roche)	TG increased in CPP; TC, HDL and LDL remained unchanged in CPP
7.	Sopher (2011) [27]	USA (New York)	cross-sectional study	PA 24	25	7.7 ± 1.4	7.2 ± 1.5	NA	NA	I	I	1.2 ± 1.1 (z-score)	1.8 ± 1.0 (z-score)	No	TC, HDL, and TG by Hitachi analyzer; LDL was calculated	TG, TC, HDL and LDL remained unchanged in PA
8.	Su (2012) [28]	Taiwan (Taichung)	cross-sectional study	CPP 249	219	8.5 ± 1.4	8.3 ± 1.4	10.4 ± 1.5	8.9 ± 2.1	134 II, 115 III	90 I, 129 II	18.7 ± 2.9	17.6 ± 3.5	No	Enzymatic color test and Olympus analyzers (AU600, Clare, Ireland)	TG and TC remained unchanged in CPP
9.	Ucar (2013) [23]	Turkey (Kayseri)	cross-sectional study	PA 69	45	7.1 ± 1.0	7.5 ± 1.9	NA	NA	NA	NA	0.5 ± 1.2 (SDS)	0.2 ± 1.1 (SDS)	NA	Integra-800 autoanalyzer (Roche)	TG and LDL decreased in PA; HDL increased in PA; TC remained unchanged in PA
10.	Hur (2017) [29]	Korea (Seoul)	cross-sectional study	CPP 164	67	8.3 ± 0.6	7.9 ± 0.6	9.7 ± 0.9	8.2 ± 0.9	II	I	0.3 ± 1.1 (SDS)	0.4 ± 0.9 (SDS)	NA	NA	TG, TC, HDL and LDL remained unchanged in CPP
11.	Zurita-Cruz (2018) [30]	México (Mexico City)	cross-sectional study	CPP 31	22	6.5 ± 2.5	6.0 ± 2.0	NA	NA	II, III	I	1.7 ± 0.4 (z-score)	1.8 ± 0.4 (z-score)	No	Colorimetric enzymatic method (Bayer Diagnostics, Puteaux, France)	TG, HDL and LDL remained unchanged in CPP
12.	Xu (2019) [24]	China (Jinan)	cross-sectional study	PT 263	222	5.3 ± 0.7	5.5 ± 0.8	6.1 ± 1.1	5.9 ± 1.2	II, III	I	15.1 ± 2.6	15.8 ± 2.1	No	AU5800 Chemistry Analyzer (Olympus, Japan)	TC, TG and LDL increased in PT; HDL remained unchanged in PT
13.	Aydin (2022) [31]	Sweden (Uppsala)	cross-sectional study	PA 48	49	7.71 ± 1.0	7.54 ± 0.9	8.2 ± 1.0	7.45 ± 1.4	NA	NA	1.2 ± 1.5 (SDS)	0.94 ± 1.6 (SDS)	No	Absorbance photometry (Roche Diagnostics, Cobas Integra 800 Device)	TC, TG, HDL and LDL remained unchanged in PA
14.	Bezen (2022) [32]	Turkey (İstanbul)	cross-sectional study	PP 35	35	8.3 ± 1.1	8.1 ± 1.0	NA	NA	NA	NA	0.34 ± 0.83 (SDS)	-0.04 ± 0.78 (SDS)	No	Spectrophotometer method in Advia 1800 device	TC, TG, HDL and LDL remained unchanged in PP

CPP-central precocious puberty; PP-premature pubarche; PA-premature adrenarche; PT-prematurethelarche; TG-triglyceride; TC-Total cholesterol; HDL-high density lipoprotein; LDL-low density lipoprotein; BMI- body mass index; GnRHa- gonadotropin releasing hormone agonist; NA- not available

### Quality assessment

Quality assessment of literature were performed according to the NOS star system, and all selected studies were scored 7 or more (Table 2).

**Table 2:** The results of NOS star system scoring

No.	Study	Adequacy of Case Definition	Representativeness of Cases	Selection of Controls	Definition of Controls	Comparability of Cases and Controls	Ascertainment of Exposure	Same Method of Ascertainment for Cases and Controls	Non-Response Rate	Total
15.	Ibáñez (1998) [43]	1	1	0	1	2	1	1	0	7
16.	Ibáñez (2004) [22]	1	1	1	1	2	1	0	0	7
17.	Teixeira (2004) [25]	1	0	1	1	2	1	1	0	7
18.	Guven (2005) [44]	1	1	0	1	2	1	1	0	7
19.	Larqué (2010) [26]	1	1	0	1	2	1	1	0	7
20.	Sorensen (2010) [45]	1	1	1	1	2	1	1	0	8
21.	Sopher (2011) [27]	1	1	0	1	2	1	1	0	7
22.	Su (2012) [28]	1	1	0	1	2	1	1	0	7
23.	Ucar (2013) [23]	1	1	1	1	2	1	1	0	8
24.	Hur (2017) [29]	1	1	0	1	2	1	0	1	7
25.	Zurita-Cruz (2018) [30]	1	1	0	1	2	1	1	1	8
26.	Xu (2019) [24]	1	1	0	1	2	2	1	0	8
27.	Aydin (2022) [31]	1	1	1	1	2	1	1	0	8
28.	Bezen (2022) [32]	1	1	0	1	2	1	1	0	8

### Meta-analysis

**Fig. 2:**
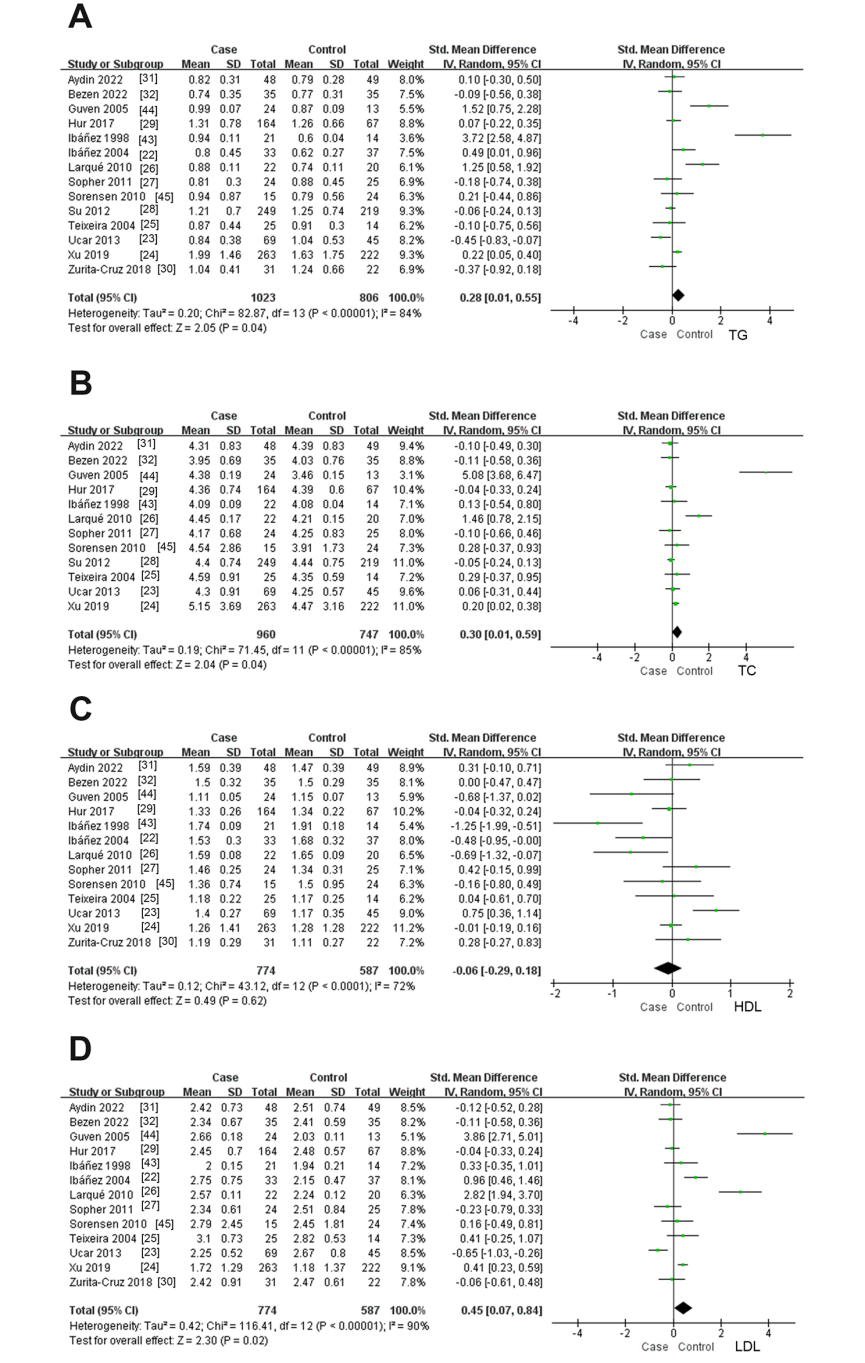
Forest plot of the levels of serum lipid profile in cases and healthy controls. Weights are from random effects analysis. (**A**) Meta-analysis of triglyceride. (**B**) Meta-analysis of total cholesterol. (**C**) Meta-analysis of high density lipoprotein. (**D**) Meta-analysis of low density lipoprotein. CI, confidence interval; SD, standard difference

Fourteen studies (*n* = 1829 participants) compared serum TG levels between girls with precocious puberty and healthy controls (Fig. 2A), and there was significant heterogeneity between studies (*I*^*2*^ = 84%; *P* < 0.00001). Precocious puberty was significantly associated with elevated serum TG levels (SMD: 0.28; 95% CI: 0.01 to 0.55; *P =* 0.04).

Twelve studies (*n* = 1707 participants) compared serum TC levels between girls with precocious puberty and healthy controls (Fig. 2B), and there was significant heterogeneity between studies (*I*^*2*^ = 85%; *P* < 0.00001). Precocious puberty was significantly associated with elevated serum TC levels (SMD: 0.30; 95% CI: 0.01 to 0.59; *P =* 0.04).

Thirteen studies (*n* = 1361 participants) compared serum HDL levels between girls with precocious puberty and healthy controls (Fig. 2C), and there was significant heterogeneity between studies (*I*^*2*^ = 72%; *P* < 0.0001). There was no significant correlation between precocious puberty and serum HDL levels (SMD: -0.06; 95% CI: -0.12 to 0.61; *P =* 0.62).

Thirteen studies (*n* = 1361 participants) compared serum LDL levels between girls with precocious puberty and healthy controls (Fig. 2D), and there was significant heterogeneity between studies (*I*^*2*^ = 90%; *P* < 0.00001). Precocious puberty was significantly associated with elevated serum LDL levels (SMD: 0.45; 95% CI: 0.07 to 0.84; *P =* 0.02).

### Results of subgroup analysis

There was significant heterogeneity between studies including meta-analysis of lipid profile levels in precocious puberty. In order to find the sources of heterogeneity and to more accurately assess the differences between girls with precocious puberty and healthy controls, subgroup analyses were conducted by disease type, region, sample size, case and control BMI differences.

In terms of disease type (Table 3), TG levels were higher in the PT group than in healthy controls (*p* = 0.01), while there was no significant difference between the CPP (*p* = 0.66), PP (*p* = 0.05) and PA (*p* = 0.58) groups, and reduced heterogeneity in CPP subgroup (*I*^*2*^ = 0%). TC levels were higher in the PT group than in healthy controls (*p* = 0.03), while there was no significant difference between the CPP (*p* = 0.66), PP (*p* = 0.22), and PA (*p* = 0.09) groups, and there was no heterogeneity in CPP subgroup (*I*^*2*^ = 0%).HDL levels were lower in PP girls compared with healthy controls(*p* = 0.03), but not significantly different in CPP (*p* = 0.98), PA (*p* = 0.32) and PT (*p* = 0.87)cases, and there was reduced heterogeneity in CPP subgroup (*I*^*2*^ = 0%). LDL levels were higher in girls with PT compared with healthy controls (*p* < 0.0001), but no significant differences in CPP (*p* = 0.86), PP (*p* = 0.05) and PA (*p* = 0.32)cases, and no heterogeneity in CPP subgroup (*I*^*2*^ = 0%).

**Table 3:** Subgroup analysis to investigate the relationship between disease type and TG, TC, HDL, LDL

Lipid parameters	Disease type			
	CPP	PP	PA	PT
**TG**				
**No. of studies**	4	5	4	1
***I*** ^***2***^	0%	91%	86%	
**SMD(95%CI)**	-0.03(-0.18, 0.11)	0.94(0.02, 1.87)	0.18(-0.47, 0.84)	0.22(0.05,0.40)
***P***	0.66	0.05	0.58	0.01
**TC**				
**No. of studies**	3	4	4	1
***I*** ^***2***^	0%	79%	94%	
**SMD(95%CI)**	-0.03(-0.18, 0.12)	0.42(-0.24, 1.09)	0.94(-0.15, 2.03)	0.20 (0.02,0.38)
***P***	0.66	0.22	0.09	0.03
**HDL**				
**No. of studies**	3	5	4	1
***I*** ^***2***^	0%	62%	76%	
**SMD(95%CI)**	0.00(-0.23, 0.24)	-0.44(-0.86, -0.02)	0.25(-0.25,0.76)	-0.01(-0.19,0.16)
***P***	0.98	0.04	0.32	0.87
**LDL**				
**No. of studies**	3	5	4	1
***I*** ^***2***^	0%	89%	94%	
**SMD(95%CI)**	-0.02(-0.26, 0.21)	0.84(0.02, 1.66)	0.56(-0.55, 1.66)	0.41(0.23,0.59)
***P***	0.86	0.05	0.32	< 0.0001

CPP-central precocious puberty; PP-premature pubarche; PA-premature adrenarche; PT-prematurethelarche; TG-triglyceride; TC-Total cholesterol; HDL-high density lipoprotein; LDL-low density lipoprotein; SMD-standard mean difference

In terms of geographical location (Table 4),TG levels were higher in European girls with precocious puberty than in healthy controls(*p* = 0.02), but not significantly different in the American(*p* = 0.18), Central Asian (*p* = 0.58) and Asian cases (*p* = 0.39), with reduced heterogeneity in the Americas (*I*^*2*^ = 0%) and Asian (*I*^*2*^ = 57%) subgroups. TC levels did not correlate with precocious puberty in all subgroups, but there was reduced heterogeneity in the American (*I*^*2*^ = 0%) and Asian (*I*^*2*^ = 53%) subgroups. HDL levels did not correlate with precocious puberty in all subgroups, but heterogeneity was reduced in the American (*I*^*2*^ = 0%) and Asian (*I*^*2*^ = 0%) subgroups. There was no correlation between LDL level and precocious puberty in all subgroups, but the heterogeneity of Americas subgroup decreased (*I*^*2*^ = 8%).

**Table 4:** Subgroup analysis to investigate the relationship between geographical location and TG, TC, HDL, LDL

Lipid parameters	Geographical location			
	Europe	Americas	Central Asia	Asia
**TG**				
**No. of studies**	5	3	3	3
***I*** ^***2***^	90%	0%	90%	57%
**SMD(95%CI)**	1.02(0.18, 1.86)	-0.23(-0.57, 0.11)	0.27(-0.67,1.21)	0.08(-0.10,0.26)
***P***	0.02	0.18	0.58	0.39
**TC**				
**No. of studies**	4	2	3	3
***I*** ^***2***^	80%	0%	96%	53%
**SMD(95%CI)**	0.42(-0.24, 1.07)	0.06(-0.36, 0.49)	1.45(-0.20, 3.10)	0.04(-0.13,0.22)
***P***	0.21	0.78	0.08	0.62
**HDL**				
**No. of studies**	5	3	3	2
***I*** ^***2***^	77%	0%	86%	0%
**SMD(95%CI)**	-0.41(-0.93, 0.11)	0.27(-0.07,0.60)	0.07(-0.71,0.84)	-0.02(-0.17,0.13)
***P***	0.12	0.12	0.87	0.78
**LDL**				
**No. of studies**	5	3	3	2
***I*** ^***2***^	90%	8%	96%	86%
**SMD(95%CI)**	0.78(-0.05, 1.61)	0.00(-0.35,0.36)	0.91(-0.76, 2.59)	0.19(-0.25,0.64)
***P***	0.07	0.98	0.28	0.39

TG-triglyceride; TC-Total cholesterol; HDL-high density lipoprotein; LDL-low density lipoprotein; SMD-standard mean difference

In terms of sample size (Table 5), the sample size < 50 group had higher TG levels than the healthy control group (*p* = 0.03), while the sample size ≥ 50 group did not differ significantly (*p* = 0.91). TC levels were higher in the sample size < 50 group than in the healthy controls (*p* = 0.03), whereas they were not higher in the sample size ≥ 50 group (*p* = 0.52), with reduced heterogeneity (*I*^*2*^ = 2%).HDL levels were not statistically different between cases and healthy controls in the sample size < 50 (*p* = 0.14) and sample size ≥ 50 (*p* = 0.38) subgroups. LDL levels were higher in girls with precocious puberty than in healthy controls (*p* = 0.03)with sample size < 50.However, serum LDL was not associated with precocious puberty in the subgroup with a sample size ≥ 50 (*p* = 0.77).

**Table 5:** Subgroup analysis to investigate the relationship between sample size, case and control BMI differences and TG, TC, HDL, LDL

Lipid parameters	Sample size		BMI difference	
	< 50	≥ 50	Yes	No
**TG**				
**No. of studies**	6	8	9	5
***I*** ^***2***^	90%	60%	90%	19%
**SMD(95%CI)**	0.99(0.08, 1.91)	0.01(-0.17, 0.19)	0.56(0.07, 1.06)	0.10(-0.07, 0.26)
***P***	0.03	0.91	0.03	0.25
**TC**				
**No. of studies**	6	6	8	4
***I*** ^***2***^	91%	2%	90%	9%
**SMD(95%CI)**	1.03 (0.08, 1.99)	0.04 (-0.07, 0.14)	0.58(0.05, 1.11)	0.10(-0.05, 0.25)
***P***	0.03	0.52	0.03	0.20
**HDL**				
**No. of studies**	6	7	8	5
***I*** ^***2***^	69%	70%	82%	0%
**SMD(95%CI)**	-0.36(-0.84, 0.12)	0.11 (-0.14, 0.36)	-0.23(-0.69, 0.24)	0.04(-0.10, 0.17)
***P***	0.14	0.38	0.34	0.60
**LDL**				
**No. of studies**	6	7	8	5
***I*** ^***2***^	92%	85%	93%	65%
**SMD(95%CI)**	1.16(0.08, 2.23)	0.05(-0.29, 0.40)	0.80(0.00, 1.61)	0.12(-0.15, 0.40)
***P***	0.03	0.77	0.05	0.38

TG-triglyceride; TC-Total cholesterol; HDL-high density lipoprotein; LDL-low density lipoprotein; SMD-standard mean difference

As for case and control BMI differences (Table 5), in the subgroup with difference, the TG levels in the cases were higher than that in healthy controls (*p* = 0.03), but not in the subgroup without difference (*p* = 0.25), the heterogeneity decreased in this subgroup (*I*^*2*^ = 19%).In the subgroup with difference, girls with precocious puberty had a higher TC level than the healthy controls (*p* = 0.03), but not in the subgroup with without difference (*p* = 0.20),while the heterogeneity was reduced in this subgroup (*I*^*2*^ = 9%). Serum HDL was not associated with precocious puberty in the subgroup with difference (*p* = 0.34) and without difference (*p* = 0.60), while there was no heterogeneity in the subgroup without difference (*I*^*2*^ = 0%). LDL level were not associated with precocious puberty in the subgroup with difference (*p* = 0.05) and without difference (*p* = 0.38).

### Sensitivity analysis

For TG, HDL and LDL levels, there was no qualitative change in the total effect size after study-by-study exclusion, indicating that this meta-analysis results were stable and reliable. For TC level, the results of this meta-analysis were weakly stable and sensitive to Guven’s study [44]. Heterogeneity was reduced after excluding this study (*I*^*2*^ from 85 to 55%), and TC levels were not statistically different between cases and healthy controls (*P* from 0.04 to 0.23).

### Publication bias

**Fig. 3:**
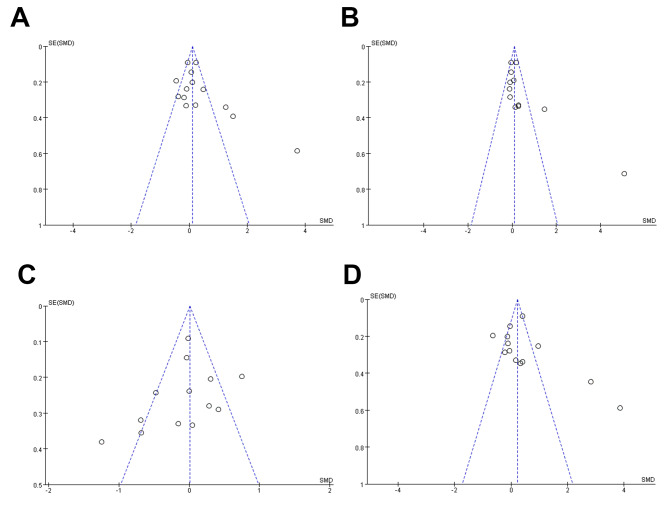
Funnel plot analysis to detect publication bias. (**A**) Funnel plot for triglyceride. (**B**) Funnel plot for total cholesterol. (**C**)Funnel plot for high density lipoprotein. (**D**) Funnel plot for low density lipoprotein. SMD, standard mean difference

Any publishing bias was found using the funnel plot method. Systematic reviewers may be at fault for publication bias if they draw final statistical findings that do not match the data by omitting previously unpublished literature. The shapes of the funnel plots were asymmetric for TG, TC, HDL and LDL, and some studies fell outside the 95% confidence interval, indicating that the asymmetry of funnel plot might be caused by the heterogeneity among the studies. This suggested that there was heterogeneity among the studies (Fig. 3).

## Discussion

A total of 14 studies were selected for this meta-analysis, with a total of 1829 girls participating. Subgroup analyses were conducted according to disease type, region, sample size, case and control BMI differences. This meta-analysis revealed significant changes in lipid levels in girls with precocious puberty: serum TG, TC and LDL levels were significantly higher in girls with precocious puberty than in healthy controls, while serum HDL levels in girls with precocious puberty were not significantly different from those in healthy controls.

Accelerated growth rates in children, especially increased fat mass, may lead to an earlier onset of puberty [46] and an increased risk of cardiometabolic disease [47], in addition, increased cardiovascular morbidity and mortality in adulthood are associated with early menarcheal age [13, 18]. Furthermore, the earlier the onset of menstruation, the greater the risk of hypertension and cardiovascular disease in adulthood [13]. Recent data, however, indicates that this relation might be mostly attributed to higher childhood obesity [19]. Early puberty causes girls to develop more quickly than girls who will experience later puberty, and as their epiphyses fuse, they seem to maintain this growth by laying down fat. Although greater adiposity may be principally responsible for the association between early maturation and higher cardiovascular risk, this does not fully explain the association [13, 14], early maturity timing per se appears to also be involved in this harmful metabolic programming [15, 20]. Girls who experience adrenarche (the rise in androgen secretion from the adrenal cortex right before puberty) earlier in life are more likely to develop insulin resistance, dyslipidemia, and obesity later in life [48]. Studies show that even in the absence of obesity, girls with PA are more likely to develop polycystic ovarian syndrome (PCOS) and have a higher future risk of developing metabolic alterations (hyperlipidemia, diabetes, and hypertension) [49]. PP, the primary clinical symptom of PA, is also associated to dyslipidemia and insulin resistance [43]. In both animal and human models, androgens are associated with changes in the distribution of body fat in females; however, gonadotropin releasing hormone agonist (GnRHa) medication does not affect this mechanism [50]. Furthermore, the prevalence of obesity and metabolic syndrome usually occurrs between the third and 50th years of life and appears to be the same in treated and untreated women with a history of CPP in childhood [51].

How dyslipidaemia causes CVD is well established. The risk of CVD is increased by hyperlipidemia, which is frequently diagnosed by elevated serum LDL levels. Furthermore, due to oxidative modification of LDL, the prevalence of atherosclerosis rises with LDL levels. As oxidised LDL accumulates in the arterial walls of the cardio-vascular region, atherosclerotic plaques gradually form. The corresponding blood vessels are blocked by the atherosclerotic plaques, which also cause atherosclerosis and raise the risk of CVD [52]. Studies have demonstrated that even if the amount of blood LDL is normal [53], partial oxidation leading toatherosclerosis cannot be excluded. Additionally, increased blood TC and TG levels are responsible for inadequate antioxidant mechanisms, which contribute to preclinical atherosclerosis [54]. These changes are therefore partly responsible for the increased incidence of CVD.

Interventions to prevent or reverse higher adiposity trajectories in girls with precocious puberty at a very young age may have significant effects on cardiovascular health in adulthood. Firstly, lipid profile should be closely monitored to detect and prevent PCOS and metabolic syndrome. Secondly, intervention to modify the lifestyle (e.g., additional diet and physical activity counseling) of the girls could prevent the long-term complications of dyslipidemia. Finally, it appears that GnRHa therapy cannot undo the undesirable body compositional alterations brought on by early maturation, inhibition of pubertal progression by GnRHa treatment is instead associated with a continual increase in obesity [55, 56]. Hence, it is worth initiating treatment with insulin sensitizers, such as metformin, which has been demonstrated to be effective in girls with precocious puberty [57, 58]. Treatment with metformin resulted in reversible reductions in TG, TC, and LDL levels as well as a significant rise in HDL level [59]. Additionally, metformin appears to have a direct impact on ovarian steroidogenesis, specifically by lowering the production of both androgen and estradiol [60, 61]. Metformin also reduces levels of plasminogen activator inhibitor type 1, known to be animportant inhibitor of fibrinolysis [62, 63]. Therefore, in order to offer the greatest promise to decrease morbidity and mortality, girls with risk factors, especially precocious puberty, should be investigated and intervened for atherosclerosis.

Subgroup analyses were conducted to further explore the sources of heterogeneity and to more accurately evaluate the association between lipid profile and precocious puberty. Heterogeneity in this meta-analysis of the correlation between TG levels and precocious puberty might be derived from disease type, region and BMI differences. The correlation between TC levels and precocious puberty might be related to disease type, region, sample size and BMI differences. The correlation between HDL levels and precocious puberty might be derived from disease type, region and BMI differences. And the correlation between LDL levels and precocious puberty might be related to disease type and region. (1) Disease types including PP, PA, PT and CPP were focused in this meta-analysis. PP, PA and PT were the variations of CPP and therefore have different degrees of impact on lipid profile levels; (2) people in different regions have different dietary habits, lifestyles and economic circumstances, which have different effects on serum lipid profile; (3) all literature included in this meta-analysis were case-control studies. If there were more cases in the case-control studies, the findings would have been influenced by more confounding factors; (4) being overweight has a significant effect on lipid profiles and therefore BMI,which is often used as a measure of total body fat,might be one of the sources of heterogeneity.

Subgroup analyses revealed partial sources of heterogeneity, but heterogeneity was still evident, so sensitivity analyses were performed. In this meta-analysis,the combined results of TC levelsassociated with precocious puberty were more sensitive to Guven’s study [44]. After excluding this reference, the heterogeneity decreased (*I*^*2*^ decreased from 85 to 55%), therefore, Guven’s study [44] had a significant impact on the heterogeneity of the combined results. After a careful reading of the literature, TC levels in the study was found to be estimated by enzyme assay method, unlike other studies with colourimetric methods or fully automated analyzers, and that other confounding factors may have contributed to the heterogeneity. The results of the sensitivity analysis showed that the combined correlation between TG, HDL, LDL levels and precocious puberty turned out to stable and reliable. In addition, there are possible signs of publication bias in this study. There is a thing called publication bias in the medical literature, it implies a higher probability of favorable results being reported. Therefore, additional thought is still required before possibly implementing the findings in clinical practice.

To our knowledge, this is the first meta-analysis to explore the relationship between lipid profile and precocious puberty. Clear eligibility criteria was developed, comprehensive search was conducted, eligibility and bias risks were both assessed, key outcomes were addressed, sensitivity and subgroup analyses were conducted, and quality assessment of literature were performed using the NOS star system. However, some limitations of this meta-analysis should be acknowledged. (1) there was significant heterogeneity between the original studies due to differences in sample size, background of study participants and methods of detecting lipid levels.The shapes of the funnel plots were asymmetric for TG, TC, HDL and LDL, and some studies fell outside the 95% confidence interval, also indicating the heterogeneity among the studies; (2) subgroup analyses of bone age and duration of precocious puberty could not be performed due to incomplete data. Instead, the heterogeneity may be related to the duration of precocious puberty or bone age, rather than physiological age per se. Because of these factors, HDL levels in girls with precocious puberty may be similar to those of healthy controls; (3) the main confounding factors affecting the lipid profiles of patients in the original study (e.g. diet, ethnicity, physical activity, etc.) were not adjusted, which might affect the conclusion of this study. So more cohort studies adjusting for these confounders are needed to justify the results of this meta-analysis; (4) the studies included in this study were all case-control studies, which would limit causal inferences–whether dyslipidemia causes precocious puberty or vice versa. Therefore, more cohort studies are needed to predict how the lipid profile develop over time in girls with precocious puberty; (5) the diagnostic criteria for identifying precocious puberty and methods for detecting lipid profile levels used in various studies varied slightly, which might also increase heterogeneity. For these reasons, we suggest that our conclusions should be taken with a grain of salt.

## Conclusion

In summary, this systematic review and meta-analysis showed that serum TG, TC and LDL levels were significantly higher in precocious puberty subjects. This implies that girls who reach sexual maturity too early can lead to some changes in lipid profile and increase the risk of developing CVD in adulthood. Thus, keeping an eye out for CVD risk markers in early-maturing girls may be a useful and effective disease prevention strategy during adolescence. We recommend that lipid profile should be assessed and comprehensively studied to ensure that early lifestyle (e.g. diet and exercise) and/or medical intervention (e.g., metformin) and to minimize the morbidity and mortality of CVD associated with dyslipidemia in girls with precocious puberty at follow-up. In addition, the relationship between serum HDL levels and precocious puberty needs to be further investigated.

### Electronic supplementary material

Below is the link to the electronic supplementary material.

Supplementary Material 1

## Data Availability

The data used to support the findings of this study are available from the corresponding author upon request.
