# Supplementary material for: Lipid profile in girls with precocious puberty: a systematic review and meta-analysis
Source: BMC Endocr Disord. 2023 Oct 18;23:225. doi: 10.1186/s12902-023-01470-8 (PMC10583444; doi:10.1186/s12902-023-01470-8)
Supplement: Supplementary file 1 — Supplementary Material 1 [file 12902_2023_1470_MOESM1_ESM.pdf]

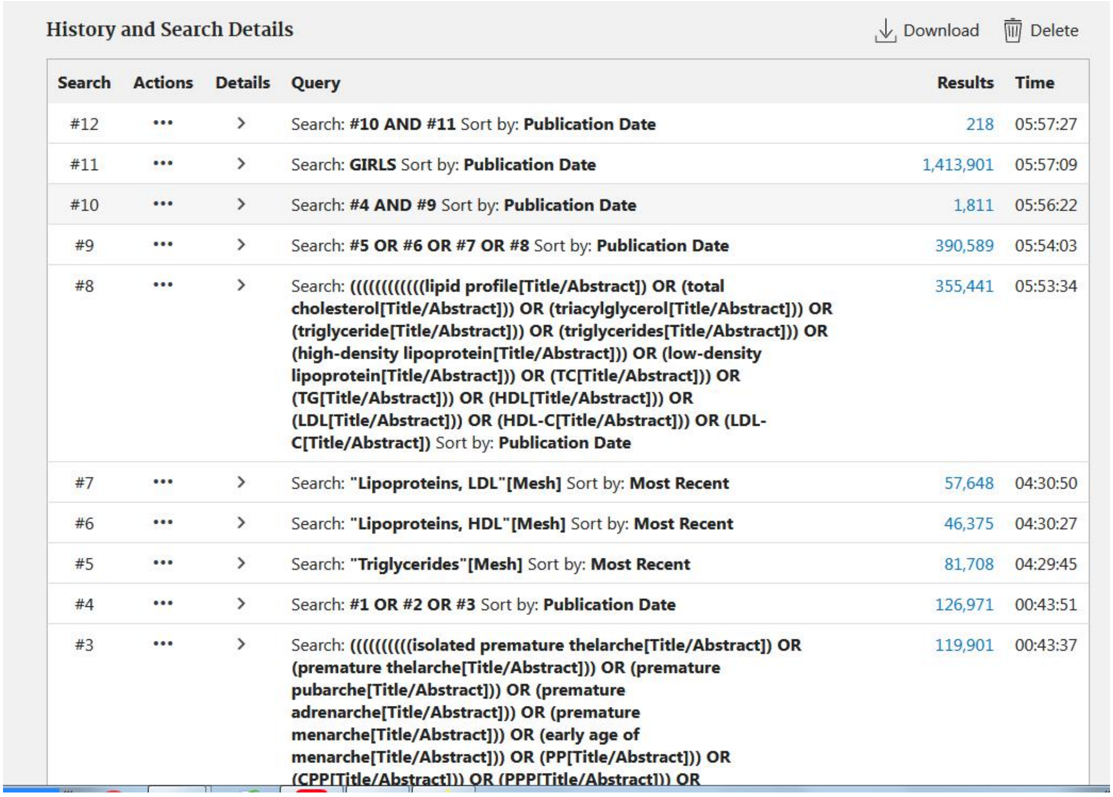

History and Search Details

Download Delete

Search	Actions	Details	Query	Results	Time
#12	•••	>	Search: **#10 AND #11** Sort by: **Publication Date**	218	05:57:27
#11	•••	>	Search: **GIRLS** Sort by: **Publication Date**	1,413,901	05:57:09
#10	•••	>	Search: **#4 AND #9** Sort by: **Publication Date**	1,811	05:56:22
#9	•••	>	Search: **#5 OR #6 OR #7 OR #8** Sort by: **Publication Date**	390,589	05:54:03
#8	•••	>	Search: **((((((((((((lipid profile[Title/Abstract]) OR (total cholesterol[Title/Abstract])) OR (triacylglycerol[Title/Abstract])) OR (triglyceride[Title/Abstract])) OR (triglycerides[Title/Abstract])) OR (high-density lipoprotein[Title/Abstract])) OR (low-density lipoprotein[Title/Abstract])) OR (TC[Title/Abstract])) OR (TG[Title/Abstract])) OR (HDL[Title/Abstract])) OR (LDL[Title/Abstract])) OR (HDL-C[Title/Abstract])) OR (LDL-C[Title/Abstract])** Sort by: **Publication Date**	355,441	05:53:34
#7	•••	>	Search: **"Lipoproteins, LDL"[Mesh]** Sort by: **Most Recent**	57,648	04:30:50
#6	•••	>	Search: **"Lipoproteins, HDL"[Mesh]** Sort by: **Most Recent**	46,375	04:30:27
#5	•••	>	Search: **"Triglycerides"[Mesh]** Sort by: **Most Recent**	81,708	04:29:45
#4	•••	>	Search: **#1 OR #2 OR #3** Sort by: **Publication Date**	126,971	00:43:51
#3	•••	>	Search: **((((((((((isolated premature thelarche[Title/Abstract]) OR (premature thelarche[Title/Abstract])) OR (premature pubarche[Title/Abstract])) OR (premature adrenarche[Title/Abstract])) OR (premature menarche[Title/Abstract])) OR (early age of menarche[Title/Abstract])) OR (PP[Title/Abstract])) OR (CPP[Title/Abstract])) OR (PPP[Title/Abstract])) OR** [illegible]	119,901	00:43:37

Figure S1 The search results in the Pubmed.

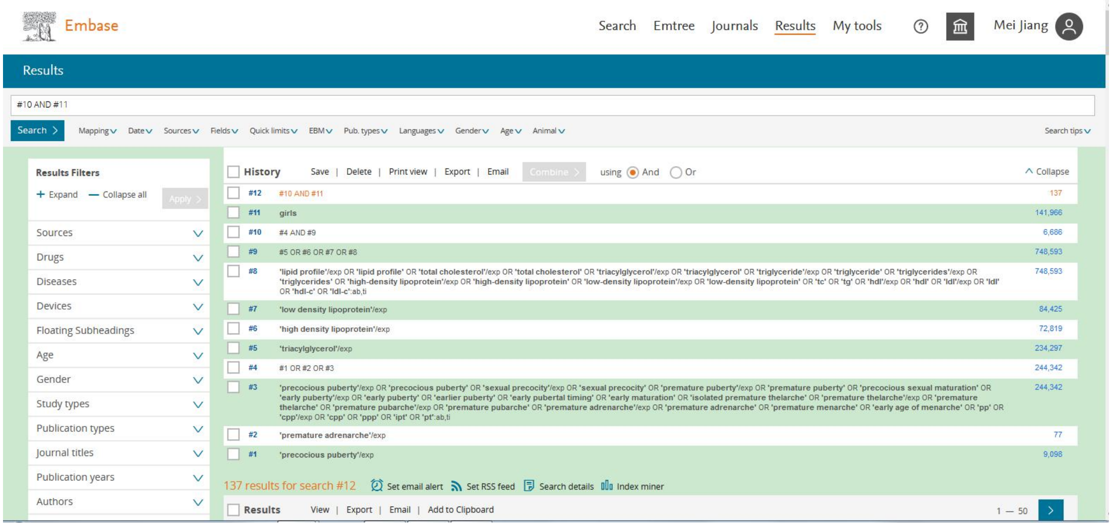

Embase

Search Emtree Journals Results My tools Mei Jiang

Results

#10 AND #11

Search Mapping Date Sources Fields Quick limits EBM Pub. types Languages Gender Age Animal Search tips

Results Filters: Expand, Collapse all, Apply; Sources, Drugs, Diseases, Devices, Floating Subheadings, Age, Gender, Study types, Publication types, Journal titles, Publication years, Authors

History | Save | Delete | Print view | Export | Email | Combine | using And Or | Collapse

#12	#10 AND #11	137
#11	girls	141,966
#10	#4 AND #9	6,686
#9	#5 OR #6 OR #7 OR #8	748,593
#8	'lipid profile'/exp OR 'lipid profile' OR 'total cholesterol'/exp OR 'total cholesterol' OR 'triacylglycerol'/exp OR 'triacylglycerol' OR 'triglyceride'/exp OR 'triglyceride' OR 'triglycerides'/exp OR 'triglycerides' OR 'high-density lipoprotein'/exp OR 'high-density lipoprotein' OR 'low-density lipoprotein'/exp OR 'low-density lipoprotein' OR 'tc' OR 'tg' OR 'hdl'/exp OR 'hdl' OR 'ldl'/exp OR 'ldl' OR 'hdl-c' OR 'ldl-c':ab,ti	748,593
#7	'low density lipoprotein'/exp	84,425
#6	'high density lipoprotein'/exp	72,819
#5	'triacylglycerol'/exp	234,297
#4	#1 OR #2 OR #3	244,342
#3	'precocious puberty'/exp OR 'precocious puberty' OR 'sexual precocity'/exp OR 'sexual precocity' OR 'premature puberty'/exp OR 'premature puberty' OR 'precocious sexual maturation' OR 'early puberty'/exp OR 'early puberty' OR 'earlier puberty' OR 'early pubertal timing' OR 'early maturation' OR 'isolated premature thelarche' OR 'premature thelarche'/exp OR 'premature thelarche' OR 'premature pubarche'/exp OR 'premature pubarche' OR 'premature adrenarche'/exp OR 'premature adrenarche' OR 'premature menarche' OR 'early age of menarche' OR 'pp' OR 'cpp'/exp OR 'cpp' OR 'ppp' OR 'ipt' OR 'pt':ab,ti	244,342
#2	'premature adrenarche'/exp	77
#1	'precocious puberty'/exp	9,098

137 results for search #12 Set email alert Set RSS feed Search details Index miner

Results View | Export | Email | Add to Clipboard 1 — 50

Figure S2 The search results in the Embase.

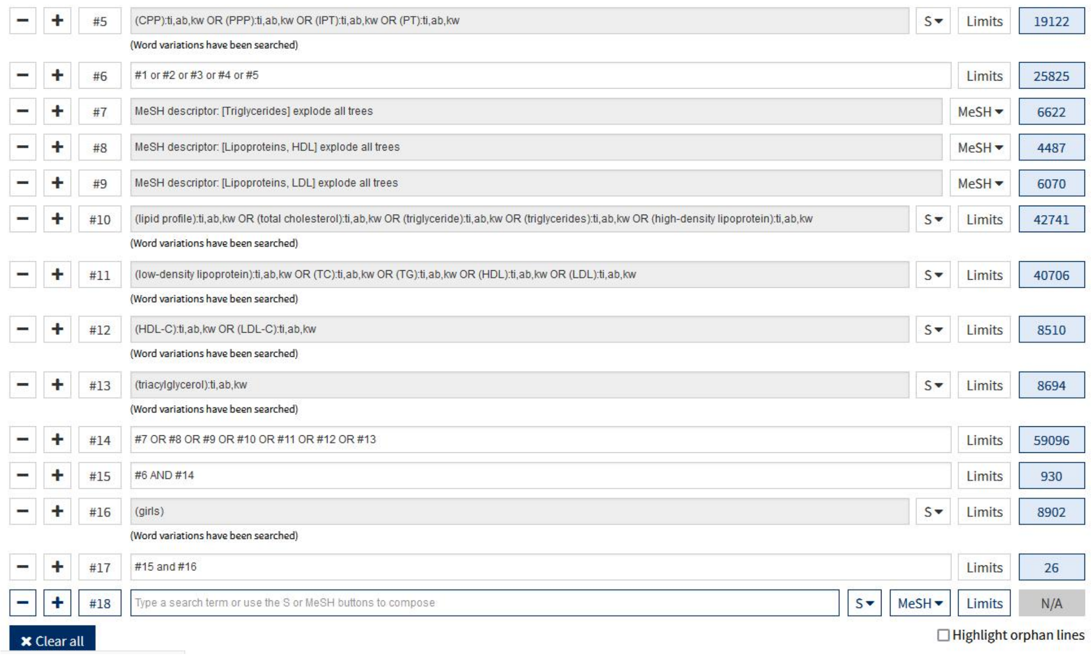

Figure S3 The search results in the Cochrane Library.

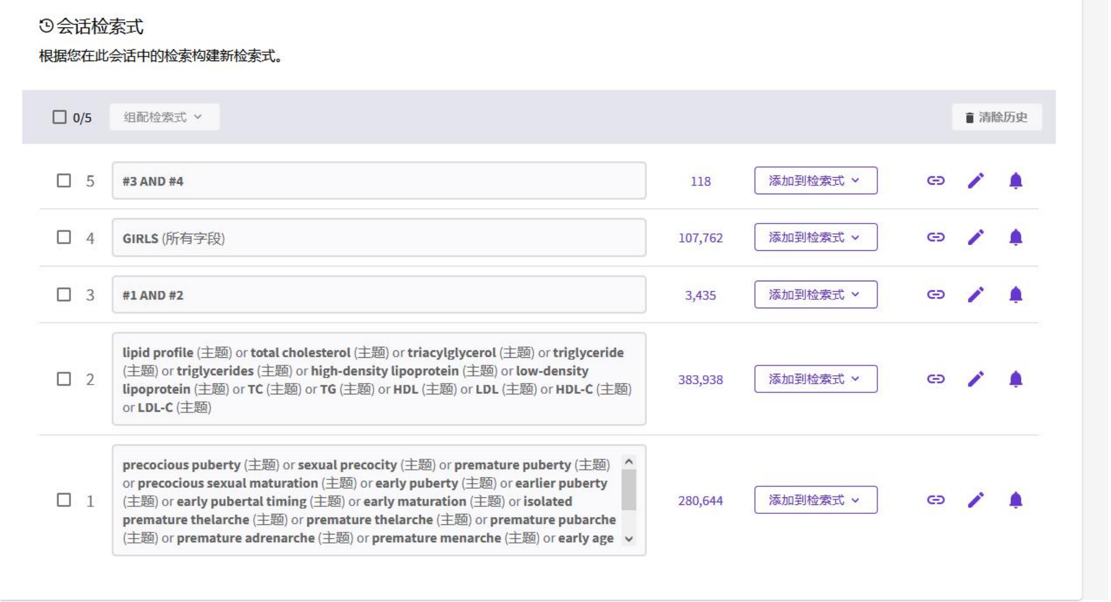

Figure S4 The search results in the Web of Science.
